# Identification of *Aphis gossypii* Glover (Hemiptera: Aphididae) Biotypes from Different Host Plants in North China

**DOI:** 10.1371/journal.pone.0146345

**Published:** 2016-01-06

**Authors:** Li Wang, Shuai Zhang, Jun-Yu Luo, Chun-Yi Wang, Li-Min Lv, Xiang-Zhen Zhu, Chun-Hua Li, Jin-Jie Cui

**Affiliations:** State Key Laboratory of Cotton Biology, Institute of Cotton Research, Chinese Academy of Agricultural Sciences, Anyang, China; French National Institute for Agricultural Research (INRA), FRANCE

## Abstract

**Background:**

The cotton-melon aphid, *Aphis gossypii* Glover (Hemiptera: Aphididae), is a polyphagous species with a worldwide distribution and a variety of biotypes. North China is a traditional agricultural area with abundant winter and summer hosts of *A*. *gossypii*. While the life cycles of *A*. *gossypii* on different plants have been well studied, those of the biotypes of North China are still unclear.

**Results:**

Host transfer experiments showed that *A*. *gossypii* from North China has two host-specialized biotypes: cotton and cucumber. Based on complete mitochondrial sequences, we identified a molecular marker with five single-nucleotide polymorphisms to distinguish the biotypes. Using this marker, a large-scale study of biotypes on primary winter and summer hosts was conducted. All *A*. *gossypii* collected from three primary hosts—hibiscus, pomegranate, and Chinese prickly ash—were cotton biotypes, with more cotton-melon aphids found on hibiscus than the other two species. In May, alate cotton and cucumber biotypes coexisted on cotton and cucumber seedlings, but each preferred its natal host. Both biotypes existed on zucchini, although the cucumber biotype was more numerous. Aphids on muskmelon were all cucumber biotypes, whereas most aphids on kidney bean were cotton biotypes. Aphids on seedlings of potato and cowpea belong to other species. In August, aphids on cotton and cucumber were the respective biotypes, with zucchini still hosting both biotypes as before. Thus, the biotypes had different fitnesses on different host plants.

**Conclusions:**

Two host-specialized biotypes (cotton and cucumber) are present in North China. Hibiscus, pomegranate, and Chinese prickly ash can serve as winter hosts for the cotton biotype but not the cucumber biotype in North China. The fitnesses of the two host-specialized biotypes differ on various summer hosts. When alate aphids migrate to summer hosts, they cannot accurately land on the corresponding plant.

## Introduction

The cotton–melon aphid, *Aphis gossypii* Glover (Hemiptera: Aphididae), is a polyphagous insect with a worldwide distribution that includes tropical, subtropical, and temperate areas [1, 2]. The species has a broad host range and can damage various crops, including those in Cucurbitaceae, Malvaceae, Solanaceae, and Rutaceae as well as some ornamental plants such as chrysanthemum [3]. The aphid causes direct physical damage by extracting carbohydrates and amino acids from plant phloem and also spreads a variety of virus diseases, such as citrus tristeza virus, watermelon mosaic virus, cucumber mosaic virus, and several viruses of potato [4], resulting in indirect losses to agricultural production. *Aphis gossypii* is thus a serious threat to many agricultural crops worldwide. In North China, the species is an important insect pest of cotton and causes yield losses by damaging cotton seedlings [5].

The life-cycle pattern of *A*. *gossypii* has been described as holocyclic in China, Japan, Korea, India, and the United States, where there have very rigorous winters [6]. The heteroecious aphids alternate between primary and secondary hosts. Their primary hosts are typically woody plants, including hibiscus, where they migrate and conduct sexual reproduction during autumn; and the secondary hosts are usually herbaceous plants, where they will have asexual reproduction (parthenogenesis) to produce numerous offspring [7, 8]. In China, cotton-melon aphid hatch from eggs on their winter hosts beginning in March and reproduce for two to three generations before alate adults migrate to summer hosts from late April to mid-May. In autumn, these morphs return to their primary hosts, where they mate with males, oviposit, and overwinter [9]. Cotton-melon aphids use more than 10 primary host species, including *Hibiscus syriacus* (hibiscus), *Punica granatum* (pomegranate), and *Zanthoxylum simulans* (Chinese prickly ash) [3, 9, 10]. In China, the most important summer hosts are cotton, cucumber, zucchini, muskmelon, potato, kidney bean, and cowpea.

Although cotton-melon aphid has a broad host spectrum, host-specialized biotypes have formed in many countries and regions through long-term evolution. A clear-cut difference in genotypes among *A*. *gossypii* from different geographical areas and host plants has been uncovered using DNA markers [11]. For example, Vanlerberghe-Masutti and Chavigny used DNA markers to separate populations of *A*. *gossypii* from Europe, Africa, and Asia into cucurbit and non-cucurbit genotypes [12]. In addition, Carletto et al. unambiguously identified five host races (Cucurbitaceae, cotton, eggplant, potato, and chili or sweet pepper) using eight microsatellites [11]. While Charaabi et al. observed strong differentiation between host plant families in Tunisia [13].

Reciprocal host transfer experiments have confirmed the existence of host-associated trade-offs in these specialized clones of *A*. *gossypii*. Guldemond et al. observed that cotton-melon aphid living on cucumber and chrysanthemum plants underwent little or no reproduction after reciprocal host transfers and developed distinct host races [14]. Liu et al. have reported that a cotton-melon aphid population in Nanjing formed host preference biotypes on cotton and cucumber plants [15]. Satar et al. contrasted life-table parameters on reciprocal hosts and then separated *A*. *gossypii* into two distinct biological groups [16]. Agarwala et al. have demonstrated that *A*. *gossypii* living on wild taro and brinjal were distinct host races with different abilities to colonize host plants, with significant differences in fecundity, intrinsic rate of increase, and net reproductive rate observed when their host plants were exchanged [17].

The purpose of this study was to identify the biotypes of cotton-melon aphid in North China and to develop a molecular method to conveniently differentiate them. Using this method and host transfer experiments, aphids from hibiscus, pomegranate, and Chinese prickly ash were identified in the spring before they transferred to summer hosts and in the autumn after they flew back to winter hosts. In addition, aphids from different summer hosts on the farm of the Institute of Cotton Research, Chinese Academy of Agricultural Sciences (CRI, CAAS), Anyang, Henan Province, China were collected for biotype identification. The general goal was to identify biotypes of *A*. *gossypii* from different winter hosts and summer hosts molecularly and by life-table analyses.

## Results

### Identification of two *A*. *gossypii*

**Fig 1:**
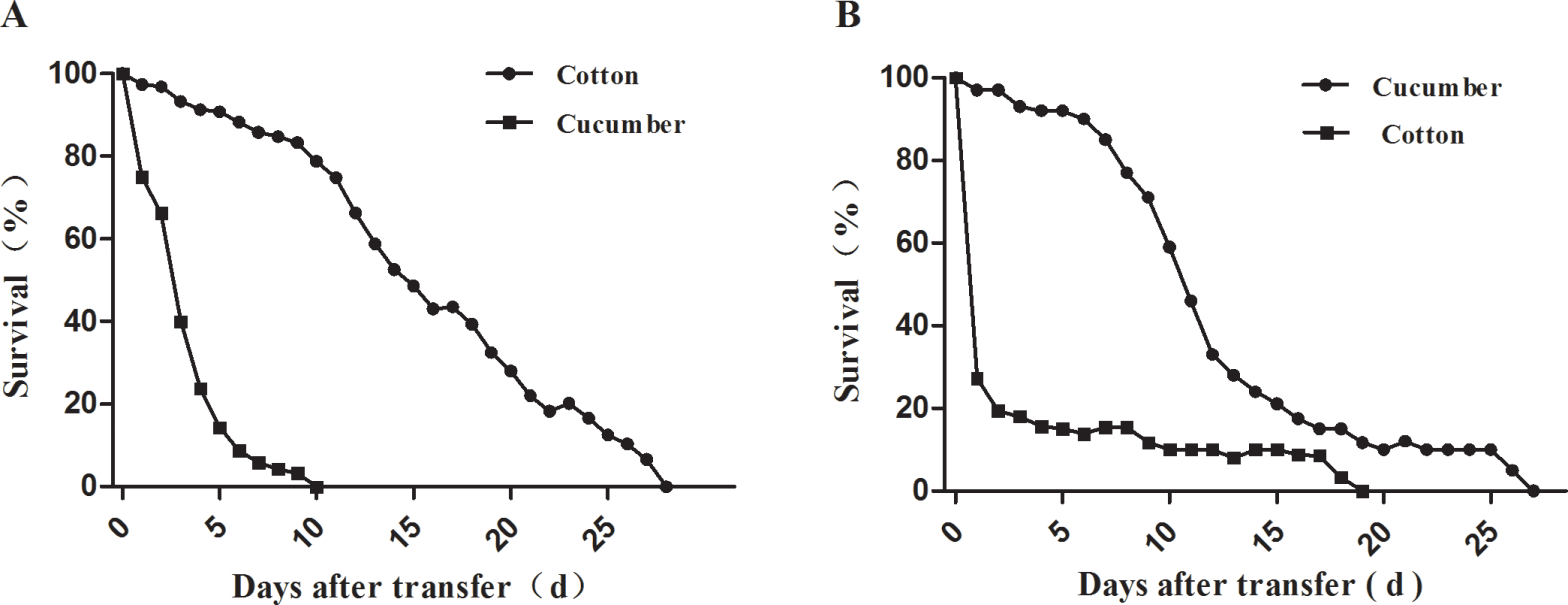
Survival curves of aphids after transfer from cotton to leaves of cotton and cucumber (A) and from cucumber to leaves of cucumber and cotton (B).

After host transfer, offspring survival rates of aphids decreased quickly. By the 4th day, only 23.8 ± 12.4% of aphids from cotton survived on cucumber (Fig 1A) and only 15.7 ± 6.2% of aphids from cucumber survived on cotton (Fig 1B), even though both biotypes survived to adulthood and reproduced on their original hosts. Although the aphids could survive for several days on the alternate host, there were significant differences in life-table parameters between hosts. The net reproductive rate *R*_*0*_ (30.7 ± 5.2) and *r*_*m*_ (0.35 ± 0.06) of the aphids from cotton on cotton were higher than those of the same aphids transferred to cucumber (0.3 ± 0.2 and −0.16 ± 0.07, respectively) (Table 1). The *R*_*0*_ of aphids from cucumber was 19.4 ± 2.2 on cucumber and 1.9 ± 0.7 on cotton, while *r*_*m*_ values were 0.35 ± 0.04 and 0.05 ± 0.04, respectively (Table 1). In summary, aphids from cotton and cucumber could not survive and establish populations on the alternate host, and there were distinct cotton and cucumber biotypes.

**Table 1:** Life-table parameters of aphids transferred from cotton to cotton and cucumber and from cucumber to cucumber and cotton leaves.

Host transfer type	Net reproductive rate *R*_*0*_	Average generation time *T*	Intrinsic rate of increase *r*_*m*_
Cotton–cotton	30.7 ± 5.2**	9.9 ± 1.5	0.35 ± 0.06**
Cotton–cucumber	0.3 ± 0.2	8.7 ± 0.7	−0.16 ± 0.07
Cucumber–cucumber	19.4 ± 2.2**	8.5 ± 1.0	0.35 ± 0.04**
Cucumber–cotton	1.9 ± 0.7	10.9 ± 1.5*	0.05 ± 0.04

Note: Values in the same column (mean ± SD) followed by “*” or “**” are significant different at the 0.05 and 0.01 level, respectively, according to the Mann–Whitney U test.

### Fitness on cotton and cucumber of *A*. *gossypii* from primary hosts

**Fig 2:**
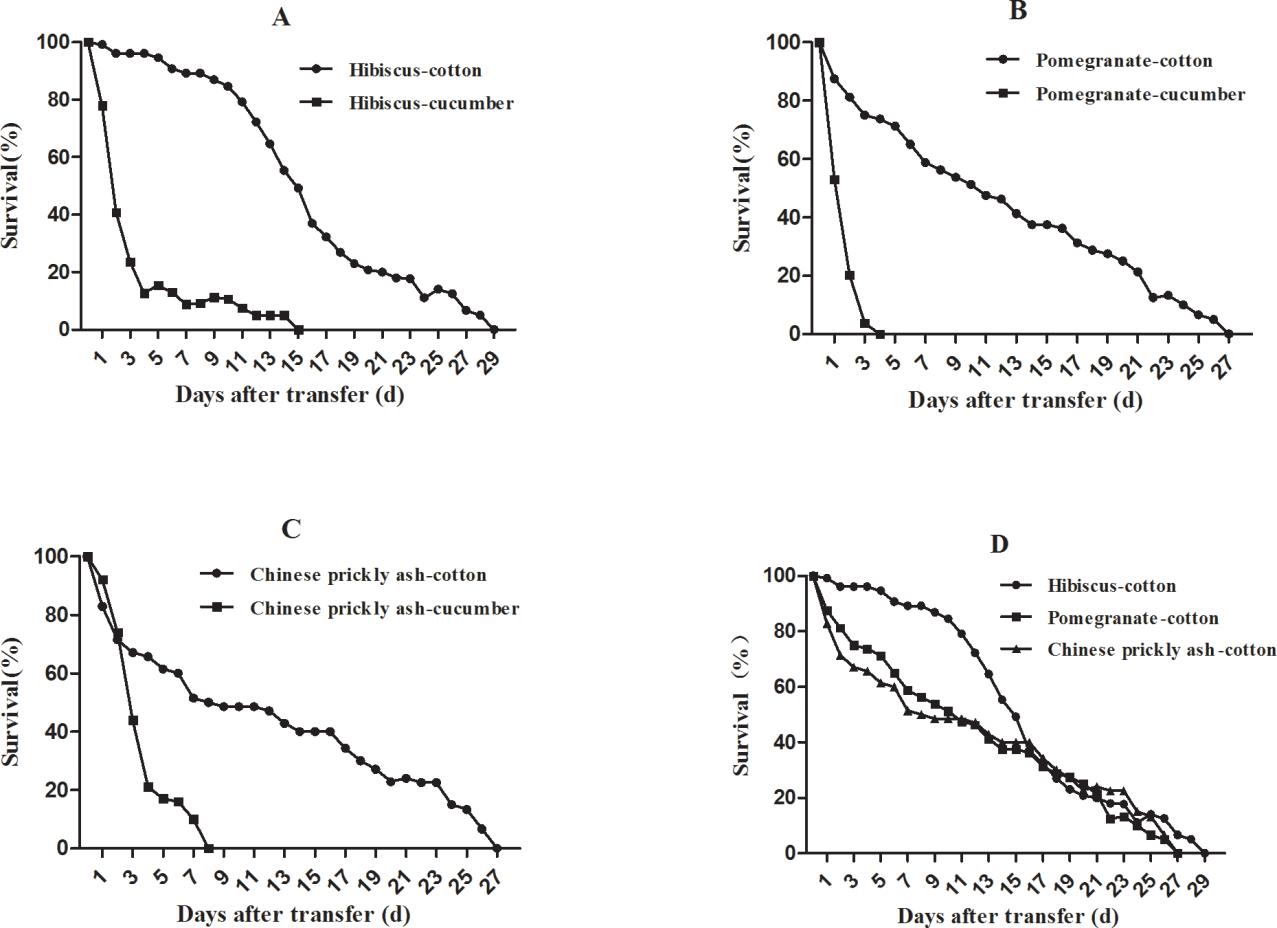
Survival curves of aphids after transfer from winter hosts to cotton and cucumber. **(**A) From hibiscus to cotton and cucumber. (B) From pomegranate to cotton and cucumber. (C) From Chinese prickly ash to cotton and cucumber. (D) From hibiscus, pomegranate and Chinese prickly ash to cotton.

After aphids were transferred from hibiscus, pomegranate, and Chinese prickly ash to cucumber, offspring survival rapidly decreased, with none ultimately none surviving. Approximately 12.9 ± 14.3%, 0%, and 21.0 ± 22.8% of aphids from the three respective hosts were alive on cucumber by the 4th day. Aphids transferred from their primary hosts to cotton were able to develop to adulthood and produce offspring (Fig 2). Significantly more aphids from hibiscus survived on cotton than did those from pomegranate and Chinese prickly ash; survival of the latter two groups did not differ significantly over the following 6 days (Table 2).

**Table 2:** Survival of aphids after transfer from winter hosts to cotton.

Host transfer type	Survival (%)					
	1 d	2 d	3 d	4 d	5 d	6 d
Hibiscus–cotton	99.2 ± 5.6a	96.2 ± 22.4a	96.2 ± 22.4a	96.2 ± 22.4a	94.6 ± 22.5a	90.8 ± 22.3a
Pomegranate–cotton	87.5 ± 13.9b	81.3 ± 15.5b	75.0 ± 13.1b	73.8 ± 11.9b	71.3 ± 12.5b	65.0 ± 10.7b
Chinese prickly ash–cotton	82.9 ± 20.6b	71.4 ± 26.7b	67.1 ± 26.9b	65.7 ± 27.6b	61.4 ± 31.3b	60.0 ± 30.0b

Note: Values in the same column (mean ± SD) followed by different letters are significantly different at P < 0.05 according to the Kruskal–Wallis test.

The life-table parameters *R*_*0*_, average generation time (*T*), and *r*_*m*_ also showed significant differences between treatments. The *R*_*0*_ of aphids transferred to cotton from hibiscus was 30.9 ± 5.9; this value was significantly higher than those of aphids from pomegranate (13.0 ± 4.3) and Chinese prickly ash (13.7 ± 7.6), which did not differ significantly from one another. Similarly, *r*_*m*_ was significantly higher for aphids from hibiscus (0.36 ± 0.06) than for aphids from pomegranate (0.22 ± 0.03) and Chinese prickly ash (0.21 ± 0.06), which did not differ significantly (Table 3). In summary, aphids transferred from pomegranate, Chinese prickly ash and especially hibiscus could survive and reproduce on cotton but not on cucumber.

**Table 3:** Life-table parameters of aphids after transfer from winter hosts to cotton.

Host transfer type	Net reproductive rate *R*_*0*_	Average generation time *T*	Intrinsic rate of increase *r*_*m*_
Hibiscus–cotton	30.9 ± 5.9a	9.6 ± 1.2b	0.36 ± 0.06a
Pomegranate–cotton	13.0 ± 4.3b	11.4 ± 0.7a	0.22 ± 0.03b
Chinese prickly ash–cotton	13.7 ± 7.6b	11.7 ± 0.6a	0.21 ± 0.06b

Note: Values in the same column (mean ± SD) followed by different letters are significantly different at P < 0.05 according to the Kruskal–Wallis test.

### Fitness of *A*. *gossypii* from cotton and cucumber on primary hosts

**Fig 3:**
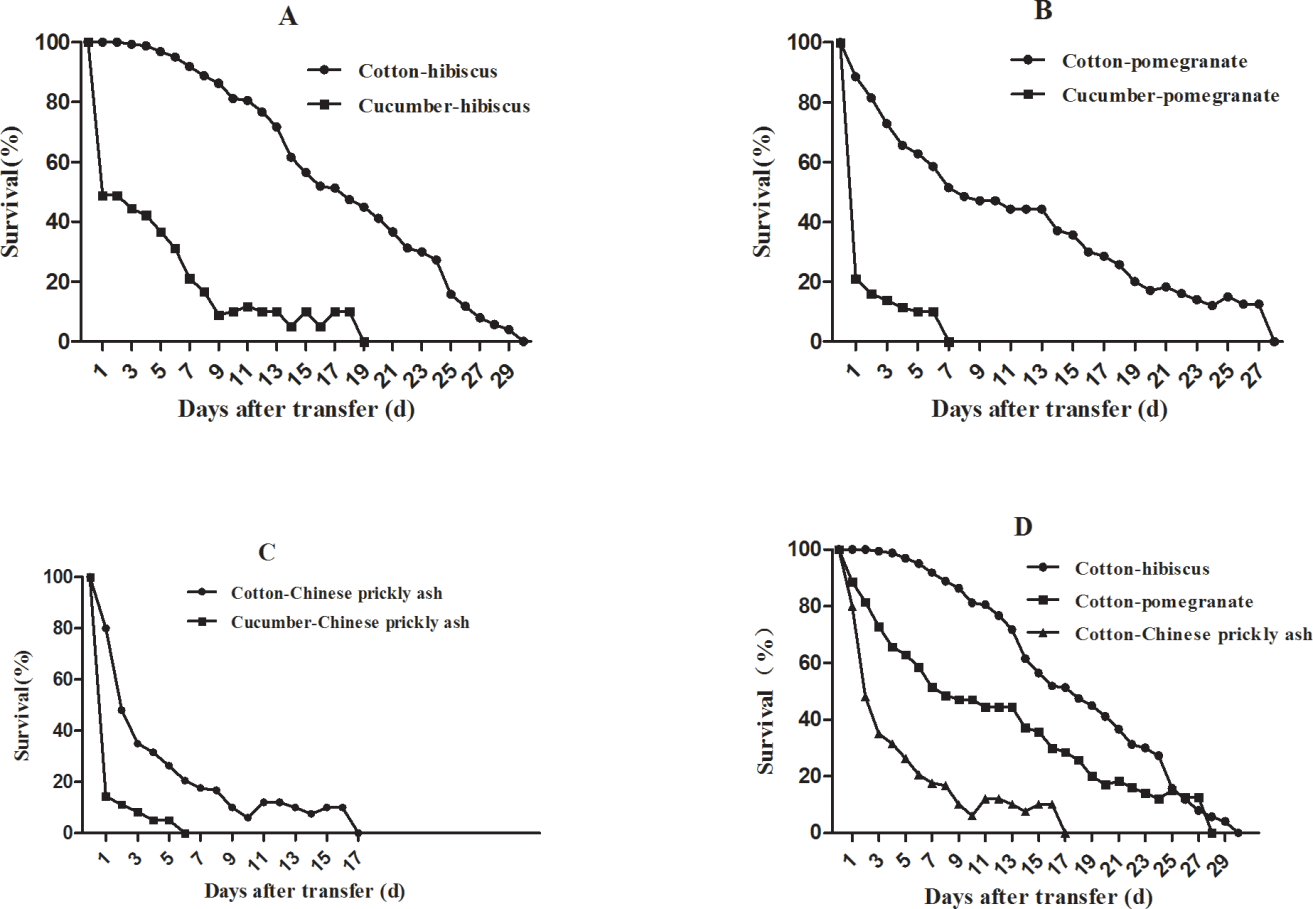
Survival curves of aphids after transfer from cotton or cucumber to winter hosts. **(**A) From cotton and cucumber to hibiscus. (B) From cotton and cucumber to pomegranate. (C) From cotton and cucumber to Chinese prickly ash. (D) From cotton to hibiscus, pomegranate and Chinese prickly ash.

After transfer of aphids from cucumber to hibiscus, pomegranate, and Chinese prickly ash, their survival rates quickly decreased to about 42.2 ± 25.8, 11.4 ± 12.1, and 5.0 ± 5.8% respectively, on the 4th day (Fig 3). Aphids transferred from cotton to hibiscus and pomegranate were able to develop to adulthood and produce offspring, whereas those shifted to Chinese prickly ash could not. Aphids from cotton after transferred to hibiscus had significantly higher survival rates than those transferred to pomegranate, with the latter having significantly higher survival rates than those transferred to Chinese prickly ash (Table 4).

**Table 4:** Survival of aphids after transfer from cotton to winter hosts.

Host transfer type	Survival (%)					
	1 d	2 d	3 d	4 d	5 d	6 d
Cotton–hibiscus	100.0 ± 0.0a	100.0 ± 0.0a	99.4 ± 2.5a	98.8 ± 3.4a	96.9 ± 6.0a	95.0 ± 9.7a
Cotton–pomegranate	88.6 ± 14.6b	81.4 ± 13.5b	72.9 ± 15.0b	65.8 ± 9.8b	62.9 ± 11.1b	58.6 ± 14.6b
Cotton–Chinese prickly ash	80.0 ± 16.5b	48.0 ± 16.7c	35.0 ± 16.1c	31.6 ± 16.1c	26.3 ± 13.4c	20.6 ± 10.0c

Note: Values in the same column (mean ± SD) followed by different letters are significantly different at P < 0.05 according to the Kruskal–Wallis test.

Among life-table parameters, *R*_*0*_ and *r*_*m*_ differed significantly for aphids transplanted from cotton to hibiscus vs. pomegranate. *R*_*0*_ values on hibiscus and pomegranate were 40.3 ± 8.9 and 6.1 ± 2.6 respectively. Similarly, *r*_*m*_ was significantly higher on hibiscus (0.38 ± 0.03) than on pomegranate (0.14 ± 0.06) (Table 5).

**Table 5:** Life-table parameters of aphids after transfer from cotton to winter hosts.

Host transfer type	Net reproductive rate *R*_*0*_	Average generation time *T*	Intrinsic rate of increase *r*_*m*_
Cotton–hibiscus	40.3 ± 8.9**	9.6 ± 0.9	0.38 ± 0.03**
Cotton–pomegranate	6.1 ± 2.6	12.9 ± 2.8 **	0.14 ± 0.06
Cotton–Chinese prickly ash	—	—	—

Note: Values in the same column (mean ± SD) followed by “*” or “**” show significantly different at the 0.05 and 0.01 level, respectively, according to the Mann–Whitney U test.

In conclusion, aphids from cotton could survive on pomegranate, Chinese prickly ash, and especially hibiscus and were able to reproduce on hibiscus and pomegranate, In contrast, aphids from cucumber did not survive on these winter hosts.

### Molecular identification of *A*. *gossypii* biotypes

**Fig 4:**
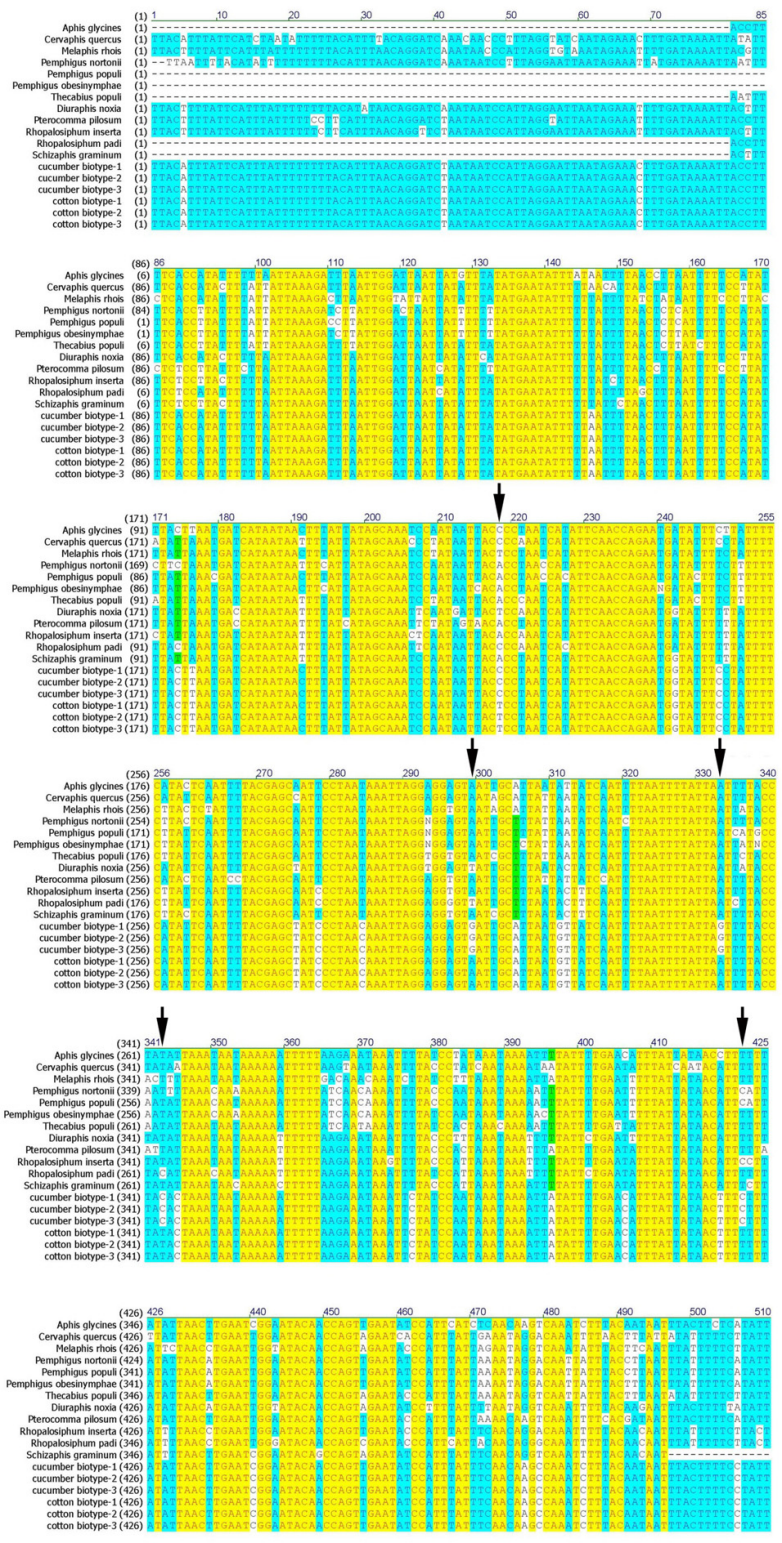
Sequence alignment of the mitochondrial DNA region used as a biotype-distinguishing molecular marker in different aphid species Arrows indicate the five single-nucleotide polymorphisms between the two host-specialized biotypes of *Aphis gossypii*. Different colored backgrounds (yellow, blue, and white) are used to highlight DNA sequence differences at each nucleotide position. Related aphid species and their corresponding GenBank accession numbers are as follows: *Aphis glycines* (KC840675.1), *Cervaphis quercus* (KF254841.1), *Melaphis rhois* (AY601894.1), *Pemphigus nortonii* (GQ284569.1), *Pemphigus populi-venae* (AY182337.1), *Pemphigus obesinymphae* (AY182345.1), *Thecabius populi-monilis* (AY182348.1), *Diuraphis noxia* (HQ528296.1), *Pterocomma pilosum* (GU457827.1), *Rhopalosiphum inserta* (AJ315894.1), *R*. *padi* (AJ315886.1), and *Schizaphis graminum* (AJ315874.1).

The complete mitochondrial genomes of two biotypes of apterous *A*. *gossypii* field-collected from cotton, cucumber and other plants were sequenced and aligned. A molecular marker with five single-nucleotide polymorphisms was identified that distinguished the biotypes. The nucleotides present at the five polymorphic positions were T, A, A, T, and T in the fragment of cotton biotype and C, G, G, C, and C in the fragment of cucumber biotype [18] (Fig 4). Identification of the *A*. *gossypii* biotypes using this method was thus simple and straight-forward.

### Biotypes of *A*. *gossypii* on primary hosts

Alate aphids were collected from hibiscus, pomegranate and Chinese prickly ash before their shift to their summer hosts in April 2014, with DNA then extracted from individual aphids. The cotton biotype represented 100% of the alates on hibiscus, 95% of the alates on pomegranate, and only 34% of the alates on Chinese prickly ash. In the case of the latter two host plants, the remaining 5% and 66% were other species of aphids, with no cucumber biotype represented (Table 6).

**Table 6:** Proportions of two cotton-melon aphid biotypes on winter hosts based on mitochondrial DNA sequences.

Collection time	Primary hosts	Alate or apterous	Total number	Cotton biotype (%)	Cucumber biotype (%)	Other species of aphids (%)
April, 2014	Hibiscus	alate	59	100	0	0
	Pomegranate	alate	41	95	0	5
	Chinese prickly ash	alate	44	34	0	66
November, 2014	Hibiscus	alate	29	72	0	28
		apterous	47	94	0	6
	Pomegranate	alate	20	20	0	80
		apterous	19	95	0	5
	Chinese prickly ash	alate	36	50	0	50
		apterous	16	44	0	56

Similar results were obtained in November 2014, with no cucumber biotype found on the three winter hosts. The cotton biotype represented 72%, 20%, and 50% of the alate aphids collected from hibiscus, pomegranate, and Chinese prickly ash, respectively, and 94%, 95%, and 44% of the apterous aphids collected about a week later (Table 6).

In summary, hibiscus, pomegranate and Chinese prickly ash were the winter hosts of the cotton biotype but not of the cucumber biotype. Moreover, hibiscus was the most suitable winter host of the cotton biotype, followed by pomegranate and then Chinese prickly ash.

### Biotypes of *A*. *gossypii* from summer hosts

Seven crops were planted on the farm in April 2014. Alate aphids were collected for sequencing in May, August, and September 2014. In May, the proportions of the cotton biotype, the cucumber biotype, and other species of aphids were respectively 33%, 2%, and 65% on cotton and 13%, 39%, and 48% on cucumber. A smaller proportion of other species of aphids was found on zucchini than on cotton and cucumber, and 37% of the aphids on zucchini were the cotton biotype and 49% were the cucumber biotype. No cotton biotype individuals were present on muskmelon, while the proportion of the cucumber biotype and other species of aphids were 76% and 24%, respectively. None of the aphids collected from potato and cowpea were *A*. *gossypii*. The aphids collected from kidney bean comprised 14% cotton biotype, 3% cucumber biotype, and 83% other species of aphids (Table 7).

**Table 7:** Proportions of two cotton-melon aphid biotypes on summer hosts based on mitochondrial DNA sequences.

Collection time	Summer hosts	Total number	Cotton biotype (%)	Cucumber biotype (%)	Other species of aphids (%)
May, 2014	cotton	40	33	2	65
	cucumber	23	13	39	48
	zucchini	43	37	49	14
	Muskmelon	17	0	76	24
	potato	44	0	0	100
	kidney bean	35	14	3	83
	cowpea	33	0	0	100
August, 2014	cotton	12	58	0	42
	cucumber	23	0	30	70
	zucchini	27	33	15	52
September, 2014	cotton	40	93	0	7
	cucumber	40	58	20	22
	zucchini	45	71	16	13

In August, there were no individuals of cucumber biotype on cotton plants nor any individuals of cotton biotype on cucumber plants. On cotton, 58% of the aphids were the cotton biotype and 42% were other species. On cucumber, 30% of the aphids were the cucumber biotype and 70% were other species. On zucchini, the proportions were 33% cotton biotype, 15% cucumber biotype, and 52% other species (Table 7).

In September, the majority of aphids on cotton (93%), cucumber (58%), and zucchini (71%) was the cotton biotype. On cotton, 7% of the aphids were other species and none were the cucumber biotype. The cucumber biotype and other species respectively represented, 20% and 22% of the aphids on cucumber and 16% and 13% of the aphids on zucchini. The proportions of the three aphid types on cotton, cucumber, and zucchini were dissimilar depending on sampling dates, with most aphids collected in September corresponding to cotton biotype. These results may be related to an outbreak of cotton biotype aphids during this period, with aphids landing randomly on other plants regardless of their suitability as hosts (Table 7).

## Discussion

### Host-specialized biotypes of *A*. *gossypii*

Biotypes represent evolutionary transitions during speciation. In insects, biotypes are most commonly defined by their ability to overcome plant resistance [19] and to use different hosts [20–22]. The evolution of biotypes is a complex process, and almost 50% of pest species with known biotypes are aphids [23]. On the basis of mitochondrial DNA divergence, Sunnuks et al. concluded that clover colonizers and lucerne colonizers were each host-restricted biotypes of *Therioaphis trifolii* and accordingly developed simple diagnostic DNA tests to distinguish between these two pest aphids [22]. Using mitochondrial DNA sequences as well, Anstead et al. confirmed the occurrence of host-adapted races in *Schizaphis graminum* clones from cultivated and non-cultivated hosts [24].

Because it can infect hundreds of plant species from numerous families, *A*. *gossypii* is considered to be a polyphagous insect [3]. This aphid uses different host species at different levels, however, and many studies have demonstrated the existence of host-specialized biotypes as highlighted by plant transfer experiments. For example, fitness has been found to be reduced when *A*. *gossypii* from Cucurbitaceae is transferred to cotton [25] or eggplant and vice versa [26]. Aphids from cotton and cucurbits cannot survive when their host plants are exchanged [14, 27, 28]. Molecular methods are also commonly used to identify biotypes of *A*. *gossypii*, as was done in a microsatellite study in north Cameroon that revealed that cotton and cucurbits were colonized by distinct groups of clonal genotypes [29].

Previous studies have shown that cotton-melon aphid in South China and the Xinjiang region can be classed into cotton and cucumber groups [15]. In North China, *A*. *gossypii* is an important pest of cotton plants, especially at the seedling stage. However, no biotype studies of *A*. *gossypii* in North China have previously been performed. In this study, we therefore used host transfer experiments and a newly developed molecular method to identify biotypes of *A*. *gossypii* from the Anyang region in central North China. Analysis of life-table parameters revealed that aphids collected from cotton and cucumber could not survive and establish a population on the alternate host. Different mitochondrial sequences were found in the two biotypes, with five single-nucleotide polymorphisms distinguishing the cotton biotype (T, A, A, T, T) from the cucumber biotype (C, G, G, C, C) [18]. Compared with other molecular methods previously applied to this aphid species, including other DNA markers and microsatellite analyses, this method was a simple way to identify *A*. *gossypii* biotypes.

### Host transfer of *A*. *gossypii*

*Aphis gossypii* has different life cycles in different countries. Throughout most of its distribution, including Europe and Africa, this aphid is considered to be anholocyclic; in other word, it reproduces continuously by apomictic parthenogenesis [3, 30]. In China, however, *A*. *gossypii* is holocyclic alternating between a usually woody primary host, such as hibiscus to which the insects migrate during autumn for sexual reproduction and usually herbaceous secondary hosts which they colonize in spring to reproduce parthenogenetically [7].

Previous studies have shown that *A*. *gossypii* from hibiscus prefers cotton to cucumber in South China [15], and that *A*. *gossypii* from *Hibiscus syriacus* and *Catalpa bignonioides* establishes populations easily on cotton but with difficulty on cucumber [28]. Our study generated similar results. Our recorded survival and life-table parameters indicate that offspring of aphids from hibiscus, pomegranate, and Chinese prickly ash can establish populations on cotton leaves but cannot survive on cucumber leaves. The cotton biotype can survive and reproduce on leaves of pomegranate, Chinese prickly ash and especially hibiscus, whereas the cucumber biotype cannot. Based on molecular markers, 100%, 95%, and 34% of the alate aphids respectively, collected from hibiscus, pomegranate, and Chinese prickly ash in April 2014 were of the cotton biotype, and none were the cucumber biotype. Results from November were similar to and lower than those from April, with cotton biotype alates representing 72% of the aphids from hibiscus, 20% from pomegranate, and 50% from Chinese prickly ash. A week later in November, the proportions of apterous aphids increased to 94% on hibiscus and 95% on pomegranate and decreased to 44% on Chinese prickly ash.

Both the molecular analysis and host transfer experiments suggested that plants of hibiscus, pomegranate, and Chinese prickly ash can serve as refuges for the cotton biotype when its summer host is no longer available, although its fitness on hibiscus is higher than on pomegranate and Chinese prickly ash, These plants do not act as refuges for the cucumber biotype, which indicates that the two biotypes have different host transfer routes. The cotton biotype migrates to summer hosts in spring and returns to the three winter hosts in autumn when the summer hosts are unsuitable. The cucumber biotype may overwinter on other, possibly wild, hosts, none of which were identified in this study.

### Host-specialized biotypes on different host plants

Some studies have reported the role of host plants in the genetic structuring of aphid populations. One of the best-documented examples of host races in aphids is the differentiation of the pea aphid, *Acyrthosiphon pisum*, into alfalfa, clover, and pea host races [31–35]. Mitochondrial DNA variations among clones of the greenbug, *Schizaphis graminum*, mirror differences in their feeding behavior, strongly supporting the existence of host-adapted races in this species [36].

Alate aphids from cotton, cucumber, zucchini, muskmelon, potato, kidney bean, and cowpea were collected for sequencing in May, August and September 2014. Cotton and cucumber biotype aphids preferred their respective hosts, with other species of aphids were also found on these two plants. Both biotypes existed on zucchini, which has been shown in previous studies to serve as a host for these types [37]. Only the cucumber biotype and other species of aphid were collected from muskmelon. Both biotypes occurred on kidney bean, although the cotton biotype was more numerous. Potato and cowpea may be unsuitable for cotton-melon aphid, as none were detected on these plants. These results indicate that cotton-melon aphid has different host-specialized biotypes. Long-term adaptation to their hosts may contribute to this differentiation.

In May, we found alate cotton biotypes on cucumber and detected cucumber biotypes on cotton. It may be that the alate aphids we collected from field crops have not undergone selective filtering by plant choice. Thus, when alate aphids migrate to their summer hosts, they do not always land immediately on their target plant.

## Conclusions

The results of the host transfer experiments show that *A*. *gossypii* in North China has two biotypes: cotton and cucumber. Hibiscus, pomegranate and Chinese prickly ash can act as winter hosts for the cotton biotype but not the cucumber biotype. A molecular identification method to differentiate the two *A*. *gossypii* biotypes was developed and used in a large-scale analysis of these biotypes on primary winter hosts and summer hosts. In May, alates of the cotton and cucumber biotypes coexisted on cotton and cucumber seedlings. Both biotypes occurred on zucchini, with more individuals of cucumber biotype than of cotton biotype inhabiting this host. Aphids on muskmelon were the cucumber biotype, whereas most on kidney bean were the cotton biotype. No *A*. *gossypii* individuals were found on potato or cowpea seedlings. In August, the aphids found on cotton and cucumber were cotton and cucumber biotypes, respectively. Both biotypes were found on zucchini, as observed earlier. Thus, the two biotypes have different fitnesses on different plants. When alate aphids migrate to their summer hosts, they do not always land on their target plant.

## Materials and Methods

### Ethics Statement

The cotton-melon aphid is a pest insect of many plant species. Because studies of *A*. *gossypii* may provide a new method to control this pest, such investigations are welcomed by farmers. No specific permits were required for the described field studies, which did not involve endangered or protected species.

### Plants and insects

After planting cotton (*Gossypium hirsutum* L.) and cucumber (*Cucumis sativus* L.) in an artificial climate chamber (26 ± 1°C, 70–80% RH, L:D = 14:10), leaves were excised from 3- to 4-week-old plants to rear aphids.

Apterous aphids used for transfers to cotton and cucumber were collected from hibiscus, pomegranate, and Chinese prickly ash in April 2014. Apterous aphids transferred to overwintering hosts were collected from cotton and cucumber on the CRI, CAAS farm (36.13°N, 114.85°E) in July 2013 and were reared on their respective host plants in growth chambers (26 ± 1°C, 70–80% RH, L:D = 14:10). Apterous aphids used for host transfer experiments between cotton and cucumber were also collected from cotton and cucumber in July 2013.

To identify host-specialized biotypes, seven common summer host species were planted in the field in April 2014: cotton, cucumber, zucchini, muskmelon, potato, kidney bean, and cowpea. Each species occupied three randomly planted 3×3 m^2^ plots. No pesticides were used during the growing period.

### Biotypes of *A*. *gossypii*

Host transfer experiments of aphids from cotton and cucumber were conducted to establish life-tables. In growth chambers (26 ± 1°C, 70–80% RH, L:D = 14:10), 5–10 apterous adult clones were introduced onto leaves of the tested host plants. After 24 h, all adult aphids were removed, and 8–10 first-stadium nymphs were kept to initiate a cohort. Thereafter, we performed daily checks on the development and reproduction of the aphids on the leaves onto which they had been transferred. Newborn nymphs were counted and then removed daily during the reproductive period. We continued the checks until all aphids died and we had recorded 10–30 replicates for each treatment [27]. Leaves used for bioassays were maintained in a fresh state in a Petri dish after wrapping the petioles with moist cotton strips; fresh leaves were replaced when needed.

Aphids on their primary hosts (hibiscus, pomegranate and Chinese prickly ash) were collected and transferred to leaves of cotton and cucumber to establish life-tables in April 2014. Aphids used to be transferred from cotton or cucumber to overwintering hosts were collected from their host plants in July 2013 and were subsequently reared on those plants; these aphids were then transferred to hibiscus, pomegranate, and Chinese prickly ash to establish life-tables in September 2014. The method used for host transfer experiments was the same as described above,with 7–24 replicates per treatment.

Life-table parameters *R*_*0*_, *T*, *r*_*m*_ were calculated for the host transfer experiment using the following equations: *R*_*0*_ = Σ*l*_*x*_*m*_*x*_, *T* = (Σ*xl*_*x*_*m*_*x*_)/(Σ*l*_*x*_*m*_*x*_), and *r*_*m*_ = ln*R*_*0*_/*T*, where *x* is age in days, *l*_*x*_ is age-specific survival, and *m*_*x*_ is age-specific number of aphid offspring [27]. Mann–Whitney U and Kruskal–Wallis tests were used to identify significance of difference between life-table parameters and survival.

### Development of a molecular identification method for *A*. *gossypii* biotypes

*Aphis gossypii* individuals possessing cotton and cucumber biotypes as confirmed by host transfer experiments were reared on their respective hosts for 40 generations. Total DNA from single individuals was extracted using a TIANamp Genomic DNA kit (Tiangen, Beijing, China) following the protocol described by the manufacturer, All extractions were stored at −20°C.

After sequencing [18], the complete mitochondrial genomes (GenBank accession number: KJ669654.1) of individuals from 20 populations (one aphid per population) of both biotypes were aligned with Vector NTI. The following primers were designed to amplify a fragment that differed consistently between the two biotypes: CytbF (5′-TACCATGAGGACAAATATCATTTTGA-3′) and 16SR (5′-AAGGGACGATAAGACCCTATAAAAC-3′). The mitochondrial DNA sequence fragment chosen to distinguish the two host-specialized biotypes was a stable region containing differential base sites that were relatively close to one another. Each PCR amplification was performed in a 50-μL volumes containing 5 μL 10× ExTaq buffer, 4 μL dNTP mixture, 2 μL of each primer, 0.25 μL ExTaq (TaKaRa, Dalian, China), 1 μL DNA template, and water. The thermocycling profile consisted of initial denaturation at 94°C for 2 min, followed by 30 cycles of 30 s at 94°C and 1 min at 64.5°C, with a final 10 min extension at 72°C. The amplified products were sequenced at Shanghai Sangon Biotech (Shanghai, China).

### Large-scale biotyping of *A*. *gossypii*

Alate aphids from winter hosts hibiscus (59 individuals), pomegranate (41), and Chinese prickly ash (44) were collected in April 2014 before they transferred to summer hosts. Alate aphids were collected from winter hosts (29, 20, and 36, respectively) in November 2014 after the aphids had returned. Apterous aphids (47, 19, and 16 from the three hosts, respectively) were collected about a week after the alates.

Alate aphids from cotton (40 individuals), cucumber (23), zucchini (43), muskmelon (17), potato (44), kidney bean (35), and cowpea (33) were collected in the field in May 2014 after their migration from winter hosts to summer hosts. Alate aphids from cotton, cucumber, and zucchini were collected in August (12, 23, and 27 individuals, respectively) and September (40, 40, and 45 respectively).

## Supporting Information

S1 Supporting Information Part of the cyt *b* base sequence of cotton and cucumber biotype aphids. (XLSX)
